# The effect of herd formation among healthcare investors on health sector growth in China

**DOI:** 10.1186/s12939-016-0393-x

**Published:** 2016-07-19

**Authors:** Zhou Lulin, Henry Asante Antwi, Wenxin Wang, Ethel Yiranbon, Emmanuel Opoku Marfo, Patrick Acheampong

**Affiliations:** School of Management, Jiangsu University, 301 Xuefu Road, Zhenjiang, Jiangsu People’s Republic of China; School of Finance and Economics, Jiangsu University, 301 Xuefu Road, Zhenjiang, Jiangsu People’s Republic of China

**Keywords:** Healthcare, Equity, Herding, Models, Stocks, Investors, China

## Abstract

**Background:**

China has become the world‘s second largest healthcare market based on a recent report by the World Health Organization. Eventhough China achieved universal health insurance coverage in 2011, representing the largest expansion of insurance coverage in human history achieved; health inequality remains endemic in China. Lessons from the effect of market crisis on health equity in Europe and other places has reignited interest in exploring the potential healthcare market aberrations that can trigger distributive injustice in healthcare resource allocation among China’s provinces. Recently, many healthcare investors in China have become more concerned about capital preservation, and are responding by abandoning long term investments strategies in healthcare. This investment withdrawal en mass is perceived to be influenced by herding tendencies and can trigger or consolidate endemic health inequality.

**Methods:**

Our study simultaneously employs four testing models (two state spaced models and two return dispersion models) to establish the existence of procyclical (herding) behavior among the stocks and its health equity implications. These are applied to a large set of data to compare and contrast results of herd formation among investors in fourteen healthcare sectors in China.

**Results:**

The study reveals that apart from the cross sectional standard deviation (CSSD) model, the remaining two models and our augmented state space model yields significant evidence of herding in all subsectors of the healthcare market. We also find that the herding effect is more prominent during down movements of the market.

**Conclusion:**

Herding behavior may lead to contemporaneous loss of investor confidence and capital withdrawal and thereby deprive the healthcare sector of the much needed capital for expansion. Thus there may be obvious delay in efforts to bridge the gap in access to healthcare facilities, medical support services, medical supplies, pharmaceuticals, biotechnology, diagnostic substances, medical laboratory and advanced medical equipment across China. Moreover, a potential crash in the healthcare market is possible in the healthcare sector as a result of persistent herding tendencies among investors and that may have more damaging consequences for health inequality in China.

## Background

According to [1] healthcare remains a very popular investment theme in developed markets especially those with ever increasing ageing population, but the potential impact is broader in Asia. Leading the Asian healthcare renaissance is China due to uninterrupted economic emancipation and intensified government’ effort to construct the social safety nets needed to reduce excessive inequality in access to healthcare in China and beyond [2]. The prevalence of health inequality in China is highlighted in several studies particularly in the recent publications of the World Health Organization, Institute for Health Economics of the Ministry of Health in China and international and regional humanitarian agencies including Medicine without Borders, the Red Cross Society etc [3]. All of these studies present a gloomy report of a national healthcare sector that is skewed in favour of the rich eastern provinces (Jiangsu, Shanghai, Guangdong, Zhejiang, Shandong etc) relative to the poorer western provinces (Tibet, Gansu, Xinjiang, Yunnan and Qinghai etc) [3].

In response to the 2011 Rio Summit on the Social Determinants of Health (that recognized a pressing need to take action to reduce health inequities), the Chinese government has augmented its ongoing health system reform (started in 2009) to improve equality, effectiveness of public health functions and rejuvenated national policies on health inequities [2, 4]. Predictably, China achieved universal health insurance coverage in 2011, representing the largest expansion of insurance coverage in human history. This success was attained owing to strong public support for government intervention in health care, renewed political commitment from top leaders, heavy government subsidies, fiscal capacity backed by China’s economic power, healthcare financing and policy responsibilities delegated to local governments and pragmatic implementation strategy for health equality [5].

The gain notwithstanding, health inequality is far from being eradicated in China. The 2015, China National Health Equality Survey (as reported by [6]) shows that the provincial disparity in infant and childhood mortality (1:5), life expectancy (3:1), maternal mortality (1:4), doctor to patient ratio (6:1), health insurance coverage (3:1) and other indicators of health inequity favoured the eastern provinces. The disparities extend beyond the provincial level to include health inequity among migrant and resident population and genders. Typical of most equality induced healthcare reforms, China’s approach to bridging the gap has largely focused on curbing disparities in health insurance coverage [7, 8], legal barriers [9], structural barriers [10], disproportionate health care financing system [11], linguistic barriers [12], health illiteracy [13], financial constrains [14], demographic factors [15] and lack of healthcare facilities and personnel [16].

However lessons from the global economic crises [17–19] and the euro debt crisis [20, 21] shows that economic indicators such as exchange rate volatility, interest rate volatility, stock market movements etc can inherently induce other forces in the healthcare market to exacerbate equity gaps or close them. In China, the healthcare market has steadily attracted a lot of attention from investors (within and outside China) eyeing countercyclical stories and demographic trends that are less affected by the vagaries of currency risk and monetary policy [22]. This is also in response to the growing opportunities in the sector as wealthier elites are willing to spend more for better healthcare [1]. Despite the initial sell-off in the early 2000s, China’s healthcare stocks as measured by the MSCI China Health Care Index bounced back substantially in mid 2002 following greater foreign investment. This was also boosted by an increase in medical supply arising out of the outbreak of zoonotic infections such as the infamous severe acute respiratory syndrome (SARS) which ravaged through China in 2003. Other factors which sustained the upsurge in healthcare stock prices over a considerable period of time include enhanced medical supply and profit from the outbreak of the pathogenic avian influenza (H5N1) in 2007 and the Haemagglutinin (1) and Neuraminidase (1) (H1N1) influenza in 2009 [23]. Thus the nearly 15 % healthcare stock return achieved between 2003 and 2012 by China was only equaled by the healthcare stocks in Japan while health-care stocks around other Asian countries remained lower or unchanged [24–26].

A 2015 report by CLSA Asia-Pacific Markets suggest that a major part of China’s healthcare sector appeal is largely because it has a lot of companies that produces drugs and medical devices that are well-positioned to tap the burgeoning domestic and global market. Additionally, China has announced an ambitious reform to provide 95 % of the population with health insurance and spend $125b (¥850b) on health-care improvements between 2015 and 2020 to stimulate demand in biopharmaceuticals and medical devices. The country also seeks to drive 25 % revenue growth in China’s pharmaceutical industry over the next two years. Unlike other sectors such as banking, telecommunications etc state-owned exert limited dominance in the healthcare markets especially the market for drug production and medical-devices in China and this is making it easier for private-sector companies to make a mark.

Another report by Templeton Asset Management issued in September 2015 indicated that healthcare sector companies such as Shandong Weigao Group Medical Polymer Co (a Hongkong maker of medical equipment such as syringes, blood bags and stents) is likely to see growth in sales with China’s medical services expand, but with additional upside from potential sales overseas. Similarly, Luye Pharma Group Ltd, a Singaporean-owned-Shanghai-listed researcher and producer of both natural drugs and chemical drugs with new formulations for use in orthopedics, neurology, gastroenterology and hepatology expects more positive returns on investments. Further, the Templeton Asset Management report posited that with the Chinese increasing their surveillance and quality control a lot of these saleable products in the domestic healthcare sector will become saleable globally and this will expand the market. Even though currently Chinese health-care stocks are generally commanding premium values to their peers in other emerging markets, a series of market fluctuations occurring intermittently (and more visibly between 2014 and 2015) in the Chinese healthcare stock market suggest that the market is susceptible to investors procyclical or herding behaviours. According to [27] a factor that recently pressured pharmaceutical and healthcare stocks in China was an announcement by China’s National Development and Reform Commission ordering price cuts for certain drugs after a government reimbursement scheme for some drugs suddenly jumped up price of healthcare products and subsequently healthcare stocks.

With this directive, healthcare stocks in China slid by about 6.4 % since the news broke in June, 2015, although the CLSA (Asia’s leading independent brokerage and investment group) allayed the fears of investors by issuing a note to claim that price control is unlikely to have any significant impact on the main listed companies and called the sell-off a buying opportunity. However, [28] argues that as China’s economic growth rate begins to tumble and the presence of strict regulations intensify, many investors both individual and institutions have become more concerned about capital preservation, and are responding by abandoning long term investments strategies in the healthcare sector. Some have also reduced the risk exposure and switched to safer asset classes with the intention of switching back as soon as the market condition improves. Bohl et. al. [29] also explain that the trend is escalating because investors are imitating each other largely due to the strong social network which has become an unrivaled heritage of the Chinese business culture.

According to [30–32] generally investors’ tendency to imitate the action of others in the economy is what the whole concept of herd or procyclical behaviour is about. This is a part or attribute of behavioural finance and economics which remains a consistently advancing field of interest in contemporary finance and economics. Understanding the ‘herding effect’ on the healthcare sector is an important issue because endemic herding tendencies can have destabilizing impacts on the healthcare sector such as price synchronicity of stocks and healthcare products and services [33, 34] and even market crash [35]. Given the magnitude of dependence on healthcare, the impacts will spread to investment, employment and equitable distribution of healthcare resources and services [21] with its attendant consequences on social stability indicators such as healthcare inequity.

Many prior studies on investor herding behavior have reported different outcomes using different methodologies such as the return dispersions among group of securities and cross sectional standard (or absolute) deviations of returns. For example contrary to the investor herding behavior based on the rational asset pricing theories and the supporting empirical work of [36] other researchers such as [37–40] found out that herding is not a significant contributor to returns on securities during market recession. These were based on studies conducted on equities in the US, international equities, commodity futures traded on European exchanges, Exchange Traded Funds and Chinese stocks respectively. Using data from sixty highly capitalized stocks, [41] used a cross sectional standard deviation study and found no evidence to suggest that foreign investors are more likely to herd in Taiwan market than domestic investors.

Weiner [42] extended the work of [43, 44] who employed cross sectional variability of factor sensitivities (instead of returns) and non-parametric methods respectively to analyse daily stock returns in South Korea. Weiner [42] rather used a hybridity of parametric and non-parametric methodologies on daily stock returns and observed that herding tendencies in crude oil and heating oil futures were not significant. These studies are inconsistent with the test of herd behavior in the domestic loan market by [45] that extended the LSV herding measure proposed by [46] within a Japanese market. On the whole, the empirical evidence of herd behavior in the extant literature provides a general indication of the fact that stock markets in general and China in particular are less informationally efficient relative to the efficient market hypothesis hence the need for a study that extends the applicability of the market efficiency hypothesis. This is because the existence of herd formation will be a clear indicator of violation of the assumptions for an efficient market in which prices fully and instantaneously reflect all available information.

Our study seeks to explore the presence of herd behavior within the healthcare industry in China due to its possible negative consequences for the healthcare market in terms of investor interest, prices of healthcare products and services which have repercussions for sustainable healthcare in China and beyond.

General studies on how herding behavior as an information aggregation mechanism influences decision making process in China is also amply represented in the current literature. For example [47] explored the neural and psychological basis of herding in purchasing books online with sixteen students selected from the Zhejiang University in China while [48] examined the influence of herd behavior in C2C e-commerce in China. The closest application of herding behavior in the healthcare sector are all outside China and are equally intended to explore its usefulness as information gathering mechanism for decision making which is sometimes at variance with the evidence based decision making process. For example [49] explores herding behavior as a psychosocial factor in quarantine during epidemic and how it can help ensure successful control while, [50] has applied herding behavior to how hospitals can form strategic groups in order to remain stable. Using a game-theoretical reflections [51] empirically validates the view that herding behavior can be used to appeal to free-riders to adopt pro-social behaviours in vaccine advocacy. Similarly, herding behavior is tested by [52] as a strategic management concept that can be used for hospital leadership and management while [53] identifies the negative aspects of herding behavior as an information aggregation system in healthcare relative to evidence based medicine. He warns healthcare institutions not to rush to implement policies and programs by merely following what contemporaries are doing. While all of the above studies have taken place outside China, these ground breaking attempts to draw on a field once thought to be purely economics and finance to the administration of mainstream healthcare is inspirational hence our attempt to advance the frontiers of the application of herding behavior in the healthcare sector by focusing on healthcare stock market and its potential implication for the whole healthcare market in China.

With respect to studies about herding behavior in the stock markets, our study is necessary in the light of limited studies on industry specific herd behavior in China and the conflicting evidence of their outcome [54] and [55] as well as the differences in the variables used in constructing and testing models [31]. To the best of our knowledge, the most recent effort at studying herding behavior in a specific industry in China are by [56] and [57] who focused on herding behavior in the housing industry in China. In both cases they employ different set of methodologies (least squares method and quantile regression method) which are totally different from those employed in this study. We extend the frontiers of current knowledge from the general (market) to the specific (industry) by empanelling and testing an ensemble of more sophisticated analytical models from the extant literature (cross sectional standard deviations-CSSD and cross sectional absolute deviations-CSAD on Chinese healthcare stock market. The results are then compared with our augmented State Space Model (which we call State Space Model 2) which is an enhancement on the existing State Space Model (which we call State Space Model I) in order to examine their respective statistical powers and subsequent robustness of inference. Understanding which models (summarized in the next section) yield herding behavior may provide information about the ways in which investors herd, the subsector with the greatest susceptibility to herding and its impact on health equity in particular and healthcare policy as a whole.

We follow the daily equally-weighted index returns data from healthcare stocks on three stock markets in China (including HongKong) namely the Shenzhen, Shanghai and Hongkong where domestic investors rather than institutional account for the highest percentage of total investment. This helps to overcome the weaknesses of using only firm level data within the market portfolio [58]. This may answer an important question posed by [59]; who expressed uncertainty as to whether herd formation would more likely occur at the level of investment in stock groups (such as stocks in an industry) where investors face similar decision problems and can observe the behaviour of others in the group (see also [60]). Further the market has seen tremendous interest by foreign investors. Because domestic individual investors usually lack the professional knowledge and skill to access accurate information easily [54, 60, 61], the resulting information asymmetry may encourage such individual investors to follow the actions of the crowd including more informed institutional and foreign investors especially in high collectivist culture such as China.

Therefore it is not out of place for one to expect the formation of investor herds in this market dominated by the less informed domestic individual investors [58, 61]. For this purpose, it is especially interesting to examine whether herd formation exists in the healthcare sector of the Chinese stock market with this unique characteristic. Following [61], we also employ two major different testing methodologies based on the dispersions of returns and factor sensitivities. The inferences are then compared from each model using a large scale data from the healthcare market in China. After presenting out data and empirical results we conclude the paper by healthcare market and policy implications before proposing future research direction.

## Methods

This study used four testing models (two state spaced models and two return dispersion models) to establish the existence of procyclical (herding) behavior among the stocks. The operationalisation of each of these models are briefly explained in the next section.

### Return dispersion models

In the return dispersion models either a cross section absolute deviation (CSSD) or a cross-sectional absolute standard deviations (CSAD) of the individual returns of the firm within groups of securities is used. The first evidence of the use of CSSD in testing herd behavior is presented by [37] who used the CSSD to measure the average proximity of returns of individual asset to the realized market average. Similarly a non-linear regression specification of the CSAD has been used by [38, 39, 62] to examine how the level of equity return dispersions relates to the overall market return. Other studies which have successfully used the CSAD approach in herd behavior measurement (albeit differences in markets) include [26, 40, 41, 60, 61]. Similar to these earlier studies, we measure return dispersion using CSSD as formulated below:

1 $$ {CSSD}_t=\sqrt{\frac{{\displaystyle \sum_{i=1}^N{\left({r}_{i,t}-{r}_{p,t}\right)}^2}}{N-1}} $$

In which case *n* represents the number of firms in the aggregate market portfolio while *r*_*i,t*_ represents the stock return observed on firm *i* for day *t* and *r*_*p,t*_ is the cross-sectional average of the *n* returns in the market portfolio for day *t*. This measure also represents the individual security return dispersion around the market average. The fundamental idea in this model is based on the argument that herd behavior prevents security returns from deviating far from the overall market return. This is because of the assumption that individuals are likely to suppress their personal beliefs and invest solely on the basis of collective actions of the market. Using the rational asset pricing models however give a different prediction that suggest that dispersions increases with changes in the absolute value of market return. This is because it is assumed that each asset is different as far as sensitivity to the market return is concerned. Additionally this model also implies that herd behavior may likely occur when market movements become extreme because they will usually go with the market consensus during such periods. Thus the behavior of the dispersion measure in (1) during periods of market stress is examined by estimating the following linear regression model:

2 $$ CSS{D}_t=\alpha +{\beta}_D{D}_t^L+{\beta}_U{D}_t^U+{\varepsilon}_t $$

where *D*_*t*_^*L*^ = 1, if the aggregate market portfolio return on day *t* lies in the *lower* tail of the return distribution; 0 otherwise, and *D*_*t*_^*U*^ = 1, if the aggregate market portfolio return on day *t* lies in the upper tail of the return distribution; 0 otherwise. Despite a concern that this may be arbitrary, this is supported in the current literature [54, 56, 59] where extreme market return is defined a return lying between one to five percent lower or upper tail of the return distribution. Moreover, the dummies in Eq. (2) seek to capture the return dispersion differences in periods of extreme market movements. Since the formation of herd behavior implies conforming to the market consensus, the presence of negative and statistically significant *β*_*D*_ (for down markets) and *β*_*U*_ (for up markets) coefficients gives an indication of herd behavior among the market participants.

The second return dispersion used in this study is proposed by [38] who also employ the cross-sectional absolute deviation of returns (CSAD) to measure return dispersion. CSAD and this is expressed as:

3 $$ CSA{D}_t=\frac{1}{N}{\displaystyle \sum_{i=1}^N\left|{r}_{i,t}-{r}_{m,t}\right|} $$

Chang et. al. [38] as reported by [61] is unconvinced about the assumptions of CAPM and suggest that return dispersions increases relative to increases in market return hence their relationship is linear. Proponents of the CAPM assumption of linearity argue that if a significant non-linear effect exist, then the results of the cross sectional standard deviations of result would not be valid. However, [38] challenges this assertion and demonstrates that in periods of economic or market stress, there is some likelihood of a non-linear relationship between return dispersions and market return (either a decline or increase). Following the example of [38] and other opponents of the CAPM assumptions, a testing model is proposed based on a general quadratic relationship between *CSAD*_*t*_ and r_*m,t*_ of the form:

4 $$ CSA{D}_t=\alpha +{\gamma}_1\left|{r}_{m.t}\right|+{\gamma}_2{r}_{m.t}^2+{\varepsilon}_t $$

With this model, the evidence of herd behavior will show by the less than proportional or low increases in the cross-sectional absolute deviation (CSAD) when there is extreme market movement. In that case herding formation will show by a negative and statistically significant non-linear coefficient γ_2_ and vice versa.

### State space models

The next model used for testing herding behavior in this study is the state space model which was first proposed by [43]. Instead of focusing on returns to measure herding behavior, the focus is rather on the cross-sectional variability of factor sensitivities. Using a single factor model based on the market return, Hwang and Salmon [43] formulated a measure of herding behavior using the relative dispersion of the betas for all assets within the market as follows:

5 $$ {E}_r\left({r}_{it}\right)={\beta}_{imt}{E}_t\left({r}_{mt}\right) $$

where *r*_*it*_ and *r*_*mt*_ are the excess returns on asset *i* and the market at time *t*, respectively, *β*_*imt*_ is the systematic risk measure, and *E*_*t*_(⋅) is conditional expectation at time *t*. In equilibrium, only β is needed to price an asset *i*. When there is herding behavior investors have no regard for equilibrium relationship of Eq. (5) hence trade to match the return of individual assets with that of the market. This gives a bias β term and expected rate of return reflecting the matching of individual asset returns with that of the market. Thus with CAPM, the presence of herding behavior means that the real β coefficient obeys Eq. (6) by replacing Eq. (5) as follows:

6 $$ \frac{E_t^b\left({r}_{it}\right)}{E_t\left({r}_{mt}\right)}={\beta}_{imt}^b={\beta}_{imt}-{h}_{mt}\left({\beta}_{imt}-1\right), $$

where *E*_*t*_^*b*^(*r*_*it*_) and *β*_*imt*_^*b*^ are the market’s biased short run conditional expectation on the excess returns of asset *i* and its beta at time *t*, and *h*_*mt*_ is a latent herding parameter that changes over time, *h*_*mt*_ ≤ 1, and conditional on market fundamentals. In general, [61, 63] argues that when (0 < *h*_*mt*_ < 1), then some degree of herding exist in the market but the intensity depends on the magnitude of *h*_*mt*_. Since the form of herding behavior being discussed is a market-wide behavior and it is assumed that Eq. (6) holds for the total assets in the market, the level of herding can be estimated with the total assets in the market instead of a single asset and this removes the effects of idiosyncratic movements in any individual *β*_*imt*_^*b*^. The standard deviation of *β*_*imt*_^*b*^ is then formulated as

7 $$ \begin{array}{l} St{d}_c\left({\beta}_{imt}^b\right)=\sqrt{E_c\left({\left({\beta}_{imt}-{h}_{mt}\left({\beta}_{imt}-1\right)-1\right)}^2\right)}\\ {}\kern5.2em =\sqrt{E_c\left({\left({\beta}_{imt}-1\right)}^2\right)}\left(1-{h}_{mt}\right)\\ {}\kern5.2em = St{d}_c\left({\beta}_{imt}\right)\left(1-{h}_{mt}\right),\end{array} $$

where *E*_*c*_(⋅) represents the cross-sectional expectation. Taking the logarithm of Eq. (7) and assuming that *Std*_*c*_(*β*_*imt*_^*b*^) can be time-varying over a time interval in response to the level of herding in the market, we rewrite Eq. (7) as

$$ \log \left[ St{d}_c\left({\beta}_{imt}^b\right)\right]={\mu}_m+{\upsilon}_{mt} $$

where *μ*_*m*_ = *E*[log[*Std*_*c*_(*β*_*imt*_)]] and *υ*_*mt*_ ∼ *iid*(0, *σ*_*mυ*_^2^). *State Space Model 1* is then estimated as

8 $$ \log \left[ St{d}_c\left({\beta}_{imt}^b\right)\right]={\mu}_m+{H}_{mt}+{\upsilon}_{mt}, $$

9 $$ {H}_{mt}={\phi}_m{H}_{mt-1}+{\eta}_{mt}, $$

where *H*_*mt*_ = log(1 − *h*_*mt*_) and *η*_*mt*_ ∼ *iid*(0, *σ*_*mη*_^2^). Equations (8) and (9) are the standard state-space model with Kalman filter estimation. In this model, the focus is basically on the dynamism in the movement patterns in the latent state variable, *H*_*mt*_, the state equation. When *σ*_*mη*_^2^ = 0, then no evidence of herding behavior is observed and that means that *H*_*mt*_ *=* 0 for all *t*. With this model a dynamic process of herding, *H*_*mt*_ is allowed to evolve over time. If the value of *σ*_*mη*_^2^ is significant, then it mean there exist herding and a significant *ϕ* implies the validity of this autoregressive structure. Alternatively [61] argues that it is possible formulate an augmented model when market volatility log *σ*_*mt*_ is added to Eq. (8) and return, *r*_*mt*_, as independent variables leading to ***State Space Model 2*** formulated as

10 $$ \log \left[ St{d}_c\left({\beta}_{imt}^b\right)\right]={\mu}_m+{H}_{mt}+{c}_{m1} \log {\sigma}_{mt}+{c}_{m2}{r}_{mt}+{\upsilon}_{mt} $$

Similarly, a significant value of *σ*_*mη*_^2^ can be interpreted as the existence of herding and a significant *ϕ* supports this particular autoregressive structure.

### Data and empirical results

The study used a dataset containing the average daily returns of 399 stocks from 14 subsectors of the healthcare industry by CLSA and trading on three stock markets namely Shenzhen, Hongkong and Shanghai between July 2004 and June 2014. Each of these stocks is further sub classified into one of 14 subcategories of the healthcare sector (healthcare facilities, medical equipment manufacture, medical supplies & distribution, pharmaceuticals diversified, pharmaceuticals generic and specialty, biotechnology, managed health care, diagnostic substances, medical laboratory and research, advanced medical equipment manufacture, healthcare transportation, therapeutic medicine, medical tourism and other medical support services) as shown in Table 1. This helps to verify whether a group is more likely to herd if trade is sufficiently homogeneous, i.e. each member faces a similar decision problem, and each member can see the trades of other group members [59]. As the years 2004, 2009 and 2014 witnessed periods of rapid foreign investment in Chinese healthcare market (because of policy incentives), data for the periods and share of foreign investment in all subsectors is highlighted. This is because the presence of foreign investors is seen as a significant precursor to herd formation [63]. The portfolio returns based on an equally weighted portfolio of all companies in the respective subsectors were then calculated. The information in Table 2 shows the summary statistics for average returns daily log, return dispersion and the average number of companies used to calculate these statistics for each sub-sector. Since the number of shares in a sector does not remain constant over time, the table shows the average number of companies during the sampling period in the second column of the table. Panel A shows that the average daily returns for all subsectors are positive. Other medical support services (4.184) and medical tourism (4.106) subsectors have the highest average daily return volatility based on the standard deviation of average daily returns. Panel B of Table 2 shows the summary statistics for daily cross sectional standard deviations within each subsector. In line with the results of Panel A, we observe higher volatility cross section in medical tourism.

**Table 1:** Shareholding percentage of market participants by sector (%)

Year	Domestic individual	Domestic institution	Foreign investor	Domestic individual	Domestic institution	Foreign investor	Domestic individual	Domestic institution	Foreign investor
	Healthcare facilities			Medical equipment manufacture			Medical supplies & distribution		
2004	90.44	0.87	7.80	90.14	0.64	9.14	81.58	0.47	15.95
2009	94.64	0.80	3.57	92.62	0.55	5.84	81.89	0.52	14.60
2014	75.42	0.91	23.67	64.99	0.90	34.11	82.54	0.54	16.93
	Pharmaceuticals diversified			Pharmaceuticals generic and specialty			Biotechnology		
2004	89.24	1.91	6.85	90.83	0.33	5.84	86.67	0.75	12.58
2009	94.74	3.17	1.09	95.64	0.72	2.64	93.29	0.40	2.31
2014	60.94	1.58	27.49	94.39	0.28	4.33	88.64	0.83	9.53
	Managed health care			Diagnostic substances			Medical laboratory and research		
2004	85.85	2.17	11.98	90.44	0.87	7.80	88.49	1.09	9.43
2009	85.98	2.97	11.05	94.64	0.80	3.57	85.87	1.47	11.67
2014	72.95	1.45	25.61	75.42	0.91	23.67	81.50	0.97	14.53
	Advanced medical equipment manufacture			Healthcare Transportation			Therapeutic Medicine		
2004	88.49	1.09	9.43	86.02	2.03	9.96	85.84	0.57	11.59
2009	85.87	1.47	11.67	89.93	1.93	6.15	90.67	0.55	7.78
2014	81.50	0.97	14.53	81.26	2.42	14.32	77.43	0.66	18.91
	Medical Tourism			Other Medical Support services					
2004	85.85	0.64	9.14	91.90	1.46	6.64			
2009	85.98	0.55	5.84	95.74	0.55	4.72			
2014	62.95	0.90	34.11	87.80	1.89	10.41

**Table 2:** Summary statistics: average daily returns and cross-sectional standard deviations

Industry	No. of firms	Observation	Mean return	Std. Dev
Panel A: average daily return				
Healthcare facilities	48	3990	0.016 %	2.457 %
Medical equipment manufacture	48	3990	0.026	2.404
Medical supplies & distribution	25	3990	0.028	2.745
Pharmaceuticals diversified	44	3990	0.014	2.774
Pharmaceuticals generic and specialty	44	3990	0.040	2.506
Biotechnology	14	3990	0.021	2.670
Managed health care	25	3990	0.045	2.476
Diagnostic substances	28	3990	0.004	4.019
Medical laboratory and research	41	3990	0.017	2.707
Advanced medical equipment manufacture	21	3990	0.044	2.827
Healthcare Transportation	4	3990	0.044	2.724
Therapeutic Medicine	9	3990	0.056	2.112
Medical Tourism	4	3990	0.056	4.106
Other Medical Support services	44	3990	0.051	4.184
Panel B: cross-sectional standard deviation				
Healthcare facilities			1.558 %	0. 911 %
Medical equipment manufacture			1.989	0.705
Medical supplies & distribution			1. 995	0.725
Pharmaceuticals diversified			2.119	0.609
Pharmaceuticals generic and specialty			2.015	0.649
Biotechnology			1.958	0.790
Managed health care			1.924	0.649
Diagnostic substances			2.199	1.094
Medical laboratory and research			1.690	0. 915
Advanced medical equipment manufacture			1.917	0.746
Healthcare Transportation			1.914	0. 944
Therapeutic Medicine			1.405	0. 999
Medical Tourism			2.444	0.920
Other Medical Support services			2.424	0.740

## Results of return dispersion models

Table 3 is the results of estimates for the CSSD base model in equation 2. Given the significant changes in dispersion and strong correlation, all estimates are made using the Newey-West heteroskedasticity and autocorrelation consistent standard errors. The MSCI China A Healthcare Index was used to represent the market and the upper and lower one and five percentile of the market return was used to represent periods of market stress. The regression output shows a negatively significant parameter estimate (β_D_) for medical tourism subsector at 99 % confidence interval. On the contrary the parameter estimates for medical equipment manufacture subsector, and specialty, managed healthcare and other medical support service is significantly positive (99 % confidence interval). The parameter estimates for pharmaceuticals generic and therapeutic medicine is significantly positive at 90 % confidence interval and the remaining sectors are positive but insignificant. Thus generally (except medical tourism) cross-section standard deviation tends to be higher during periods of extremely down markets. This suggests that the included stocks do not move in the same direction as the market during periods of market stress but rather deviate more from general market movements when the market takes an extreme fall. Consistent with the work of [62], this observation does not support herding.

**Table 3:** Regression coefficients for *CSSD*_*t*_ = *α* + *β*_*D*_ *D*_*t*_^*L*^ + *β*_*U*_ *D*_*t*_^*U*^ + *ε*_*t*_ (R^2^-values in parentheses)

Return dispersions	Market return in the extreme upper/lower 1 % of the return distribution			Market return in the extreme upper/lower 5 % of the return distribution		
*Subsector*	α	β_D_	β_U_	α	β_D_	β_U_
Healthcare facilities	1.554 %	0.145 %	0.194 %**	1.527 %	0.454 %***	0.169 %^***^
		(0.586)	(0.649)		(0.573)	(0.724)
Medical equipment manufacture	1.857	0.699***	0.442***	1.724	0.541***	0.451***
		(0.830)	(0.828)		(0.979)	(0.681)
Medical supplies & distribution	1.794	0.167	0.026	1.757	0.497***	0.160***
		(0.549)	(0.520)		(0.570)	(0.524)
Pharmaceuticals diversified	2.116	0.146	0.149*	2.090	0.449***	0.142***
		(0.669)	(0.596)		(0.738)	(0.665)
Pharmaceuticals generic and specialty	2.009	0.144*	0.404***	1.970	0.402***	0.175***
		(0.684)	(0.549)		(0.949)	(0.693)
Biotechnology	1.955	0.079	0.016	1.927	0.422***	0.147***
		(0. 638)	(0.742)		(0.561)	(0.563)
Managed health care	1.919	0.479***	0.044	1.790	0.476***	0.194***
		(0.915)	(0.671)		(0.563)	(0.899)
Diagnostic substances	2.196	0.079	0.176	2.162	0.441***	0.404***
		(0. 908)	(0.695)		(0.539)	(0.921)
Medical laboratory and research	1.691	−0.0002	−0.074	1.670	0.407***	0.099
		(0.826)	(0.750)		(0.570)	(0.537)
Advanced medical equipment manufacture	1.914	0.076	0.165	1.771	0.404***	0.414***
		(0.827)	(0.619)		(0.599)	(0.636)
Healthcare Transportation	1.916	0.061	−0.119*	1.776	0.456***	0.114*
		(0.612)	(0.853)		(0. 618)	(0.808)
Therapeutic Medicine	1.495	0.492*	0.569***	1.467	0.479***	0.175***
		(0.971)	(0.520)		(0.798)	(0.971)
Medical Tourism	2.440	−0.479***	−0.475***	2.419	0.069	−0.012
		(0.949)	(0.911)		(0.736)	(0.568)
Other Medical support services	2.067	0.447***	0.110***	2.041	0.417***	0.121***
		(0.806)	(0.663)		(0.793)	(0.949)

(***, ** and * denote statistical significance at 1, 5 and 10 % respectively)

In Table 4 the estimates for the model based CSAD non-linear equation 4 has been presented. Similar to [38], three distinct regressions were run for each subsector: one that uses the entire sample, and two limiting the data to up (or down) movement of the market index. Running separate models in this way allows us to examine if there is any asymmetric effect of herd behavior. The results from the non-linear model give completely different results than the first method. Table 4 again shows that the first nonlinear term (γ2) is statistically significant in almost all subsectors. It is very instructive to note that unlike the linear model, the R^2^ values of the nonlinear regression models are generally higher than those of the linear model. This indicates that the non linear regression fits better than the linear regression in Table 3 as the proportion of the variance in herding that is predictable from the independent variable is higher. Generally, the information indicates that the inference made from the linear model disclosed in Table 3 may be spurious. Thus we rely heavily on evidence from the nonlinear test in Table 4 to make inferences about herding. In that regard, evidence of herd formation is found in all the subsectors and regressions yield statistically significant and negative estimates γ_2_ which indicates a declining non-linear relationship between the market return and equity return dispersions. The results of regression analysis performed with respect to up and down markets separately show herding effect is mostly prominent in market losses. The results suggest that herd formation is most likely observed during periods of market losses.

**Table 4:** Regression coefficients for *CSAD*_*t*_ = *α* + *γ*_1_|*r*_*m. t*_| + *γ*_2_ *r*_*m. t*_^2^ + *ε*_*t*_ (R^2^-values in parentheses)

Absolute deviation	Whole sample			Down market (R_m_ < 0)			Up market (R_m_ > 0)		
Industry	α	γ_1_	γ_2_	α	γ_1_	γ_2_	α	γ_1_	γ_2_
Healthcare facilities	1.016	0.162***	−0.017***	1.002	0.117***	−0.029***	1.046	0.095**	−0.004
		(.799)	(.534)		(.831)	(.727)		(.695)	(.856)
Medical equipment manufacture	1.196	0.194***	−0.014**	1.174	0.165***	−0.024***	1.121	0.117***	0.0004
		(.860)	(.841)		(.849)	(.835)		(.802)	(.823)
Medical supplies & distribution	1.111	0.404***	−0.044***	1.177	0.471***	−0.056***	1.146	0.121***	−0.029***
		(.789)	(.685)		(.895)	(.869)		(.827)	(.820)
Pharmaceuticals diversified	1.494	0.129***	−0.040***	1.470	0.171***	−0.049***	1.421	0.169***	−0.017***
		(.672)	(.674)		(.851)	(.812)		(.813)	(.821)
Pharmaceuticals generic and specialty	1.424	0.106***	−0.022***	1.407	0.157***	−0.042***	1.445	0.144***	−0.009
		(.725)	(.851)		(.703)	(.805)		(.668)	(.811)
Biotechnology	1.196	0.195***	−0.027***	1.177	0.157***	−0.049***	1.421	0.122***	−0.014
		(.722)	(.847)		(.802)	(.637)		(.804)	(.773)
Managed health care	1.144	0.142***	−0.040***	1.109	0.420***	−0.042***	1.160	0.161***	−0.017***
		(.703)	(.840)		(.822)	(.645)		(.731)	(.819)
Diagnostic substances	1.545	0.101***	−0.024**	1.551	0.146***	−0.044**	1.544	0.145***	−0.009
		(.846)	(.824)		(.665)	(.725)		(.696)	(.822)
Medical laboratory and research	1.124	0.104***	−0.046***	1.105	0.157***	−0.044***	1.144	0.146***	−0.027***
		(.837)	(.811)		(.736)	(.666)		(.773)	(.766)
Advanced medical equipment manufacture	1.154	0.141***	−0.029***	1.155	0.402***	−0.044***	1.155	0.149***	−0.011
		(.660)	(.875)		(.748)	(.812)		(.768)	(.812)
Healthcare Transportation	1.147	0.171***	−0.045***	1.141	0.447***	−0.057***	1.166	0.194***	−0.044***
		(.860)	(.651)		(.806)	(.805)		(.745)	(.725)
Therapeutic Medicine	0.924	0.125***	−0.007	0.771	0.124***	−0.027***	0.977	0.002	0.020**
		(.894)	(.749)		(.783)	(.839)		(.637)	(.762)
Medical Tourism	1.597	0.175***	−0.057***	1.577	0.194***	−0.060***	1.617	0.157***	−0.057***
		(.564)	(。627)		(.807)	(.890)		(.757)	(.826)
Other Medical support services	1.446	0.192***	−0.017***	1.426	0.147***	−0.024***	1.465	0.147***	−0.012*
		(.590)	(.628)		(.636)	(.642)		(.734)	(.857)

(***, **, and * denote statistical significance at 1, 5, and 10 %, respectively)

### Results of state space models

The information in Table 5 presents the results of the ***State Space Model 1***. The results show strong evidence of herding through *H*_*mt*_ and this is consistent with the findings using the non-linear model. It is observed that *H*_*mt*_ is highly persistent with large and significant values of ${\widehat{\phi}}_m$. Moreover *σ*_*mη*_ estimates are highly significant and indicate herd behavior. On the other hand, Table 6 show results of the augmented ***State Space Model 2*** which contains two market variables (market return and market volatility). If these two are added in the measurement equation, it allows us to analyze the degree of herding, given the state of the market. In that regard, the findings are similar to that of the ***State Space Model 1*** shown in Table 5.

**Table 5:** State space model 1 (R^2^-values in parentheses) log[*Std*_*c*_(*β*_*imt*_^*b*^)] = *μ*_*m*_ + *H*_*mt*_ + *υ*_*mt*_ and *H*_*mt*_ = ϕ_*m*_ *H*_*mt* − 1_ + *η*_*mt*_

Industry	*μ*_*m*_	*ϕ*_*m*_	*σ* ^2^_*mυ*_	*σ* ^2^_*mη*_
Healthcare facilities	−0.675***	0.940***	0.075***	0.099***
	(.760)	(.791)	(.817)	(.549)
Medical equipment manufacture	−0.645***	0.940***	0.019***	0.091***
	(.901)	(.249)	(.543)	(.651)
Medical supplies & distribution	−0.570***	0.945***	0.047***	0.091***
	(.861)	(.521)	(.672)	(.621)
Pharmaceuticals diversified	−0.647***	0.957***	0.040***	0.067***
	(.747)	(.894)	(.617)	(.549)
Pharmaceuticals generic and specialty	−0.697***	0.914***	0.054***	0.114***
	(.651)	(.913)	(0.572)	(.781)
Biotechnology	−1.456***	0.927***	0.015***	0.064***
	(.710)	(.592)	(.169)	(549)
Managed health care	−0.657***	0.954***	0.025***	0.071***
	(.628)	(.084)	(.095)	(.067)
Diagnostic substances	−0.577***	0.919***	0.072***	0.142***
	(.801)	(.667)	(.074)	(.588)
Medical laboratory and research	−0.709***	0.792***	0.070***	0.165***
	(.570)	(.595)	(.684)	(.762)
Advanced medical equipment manufacture	−0.707***	0.949***	0.047***	0.104***
	(.590)	(.748)	(.656)	(.565)
Healthcare Transportation	−0.727***	0.905***	0.100***	0.165***
	(.755)	(.656)	(.524)	(.671)
Therapeutic Medicine	−0.457***	0.920***	0.022***	0.072***
	(0.531)	(.675)	(4.400)	(.893)
Medical Tourism	−1.172***	0.997***	0.047***	0.054***
	(.741)	(.667)	(.523)	(.974)
Other Medical support services	−1.474***	0.916***	0.075***	0.091***
	(.518)	(.571)	(.732)	(.591)

(***, **, and * denote statistical significance at 1, 5, and 10 %, respectively)

**Table 6:** State space model 2 (R^2^-values in parentheses) log[*Std*_*c*_(*β*_*imt*_^*b*^)] = *μ*_*m*_ + *H*_*mt*_ + *c*_*m*1_ log *σ*_*mt*_ + *c*_*m*2_ *r*_*mt*_ + *υ*_*mt*_ and *H*_*mt*_ = ϕ_*m*_ *H*_*mt* − 1_ + *η*_*mt*_

Industry	*μ*_*m*_	*ϕ*_*m*_	*σ* ^2^_*mυ*_	*σ* ^2^_*mη*_	log *σ*_*mt*_	*r*_*mt*_
Healthcare facilities	0.451***	0.941***	0.075***	0.097***	0.659***	−0.005
	(.569)	(.542)	(0.903)	(.820)	(.659)	(.580)
Medical equipment manufacture	0.879***	0.941***	0.019***	0.091***	0.672***	0.005
	(.816)	(.509)	(.785)	(.690)	(.053)	(.614)
Medical supplies & distribution	−0.747***	0.944***	0.047***	0.091***	−0.151***	0.046
	(.739)	(.600)	(.0523)	(.875)	(.532)	(.518)
Pharmaceuticals diversified	0.897***	0.957***	0.040***	0.067***	0.776***	0.027
	(.846)	(.697)	(.817)	(.647)	(.557)	(.657)
Pharmaceuticals generic and specialty	−0.706***	0.914***	0.054***	0.114***	−0.057***	−0.069
	(.847)	(.684)	(.710)	(.567)	(.769)	(.739)
Biotechnology	1.447***	0.917***	0.015***	0.064***	1.657***	0.047
	(.542)	(.910)	(.812)	(.536)	(.566)	(.588)
Managed health care	−1.627***	0.950***	0.024***	0.072***	−0.545***	0.026
	(.814)	(.596)	(.640)	(.714)	(.984)	(.717)
Diagnostic substances	1.440***	0.917***	0.072***	0.142***	1.092***	−0.067
	(.586)	(.635)	(.671)	(.812)	(.938)	(.814)
Medical laboratory and research	−2.479***	0.792***	0.070***	0.164***	−0.970***	−0.092
	(.701)	(.598)	(。673)	(.530)	(.633)	(.636)
Advanced medical equipment manufacture	−1.470***	0.945***	0.047***	0.104***	−0.429***	0.029
	(.782)	(.609)	(.795)	(.618)	(.598)	(.814)
Healthcare Transportation	−0.471***	0.907***	0.100***	0.165***	0.111***	0.006
	(.657)	(.740)	(.561)	(.767)	(.840)	(.733)
Therapeutic Medicine	−0.541***	0.919***	0.021***	0.072***	−0.047***	0.017
	(.556)	(.592)	(.542)	(.642)	(.870)	(.543)
Medical Tourism	0.471	0.997***	0.047***	0.054***	1.022*	0.007
	(.748)	(.538)	(.640)	(.539)	(.529)	(.626)
Other Medical support services	−0.547***	0.954***	0.074***	0.095***	0.014	0.147
	(.512)	(.719)	(.976)	(.529)	(.944)	(.727)

(***, **, and * denote statistical significance at 1, 5, and 10 %, respectively)

Further, if the level of market volatility and market return are accounted for, the term *H*_*mt*_ remains significant when the two explanatory variables are included. The findings indicate that herding behavior could explain changes in the volatility of factor sensitivities or dispersion of the betas for all assets in the market (*Std*_*c*_(*β*_*imt*_^*b*^), instead of changes in fundamentals. Moreover positive and significant coefficients for the term log *σ*_*mt*_ were estimated for most of the stocks in subsectors in the healthcare industry and that indicates an increasing dispersion of the betas for all assets in the market (*Std*_*c*_(*β*_*imt*_^*b*^) with market volatility. This result is equally consistent with the previous studies that suggest that herding is more likely to occur during periods of market stress, i.e. highly volatile periods. Similarly, Table 6 shows that subsectors such as healthcare facilities, medical equipment manufacture, pharmaceuticals diversified, biotechnology, diagnostic substances and healthcare transportation sub sectors have dispersion of the betas for all assets in the market (*Std*_*c*_(*β*_*imt*_^*b*^) that increases as market volatility rises since the coefficients for the market volatility term (log *σ*_*mt*_) have significant and positive values. It is worth noting from Table 1 that that these subsectors (healthcare facilities, medical equipment manufacture, pharmaceuticals diversified, biotechnology, diagnostic substances) are among those that have seen increase in foreign shareholding presence between 2004 and 2014. This gives credence to the view of [63, 64] that foreign investors may exhibit herding behavior more visibly than domestic individual investors. Comparing the estimates for ${\widehat{\phi}}_m$ across subsectors, it is noted that the effect of herd behavior in medical tourism and other medical support services are relatively higher than the other subsectors. From Table 1 it is observed that the medical tourism and other medical support services have seen significant increase in foreign investors since 2004 and this can be the source of the herding behavior.

## Discussion of findings

We sought to extend testing of herd behavior of investors to the healthcare stock market in China based on firm-level data on several healthcare related enterprises operating in 14 subsectors of the healthcare market. In addition, we simultaneously employ different models of herding at a large set of data to compare and contrast results to contextually test how these different approaches yield different or similar results of herd formation among investors. We found most of the models yielding evidence of endemic herding behavior in most of the healthcare subsectors in China. As indicated in the introductory section, there is enormous literature that supports the destabilizing effect of herd behavior in a sector including the healthcare sector. For example the effect of ‘herding’ on stock price synchronicity as suggested by [33, 34, 63] threatens distributive justice in healthcare resources in the sense that herding tendencies can stimulate sharp fluctuation in the prices of healthcare stocks en masse.

This may lead to contemporaneous loss of investor confidence and capital withdrawal (see Akerlof and Shiller’s revival of Keynes’ discussion of the “animal spirits” that animate economic behavior) and thereby deprive the healthcare sector of the much needed capital for expansion. Thus there may be obvious delay in efforts to bridge the gap in access to healthcare facilities, medical support services, medical supplies, pharmaceuticals, biotechnology, diagnostic substances, medical laboratory and advanced medical equipment across China. Consistent with the work of [5] this may be a major challenge in China where inner (western) and some northern provinces, rural areas, and communities with high concentrations of minority populations have limited access to medical care due to the scarcity of primary care practitioners, facilities, medical supplies, poor transportation and poor communication.

Evidence of this challenge may be inferred from the decisions of renowned listed healthcare companies such as Jiangsu Hengrui Med Co, Kangmei Pharmaceutical, Yunnan Baiyao Group Co, Dong E E Jiao Co, Tasly Pharmaceutical, Shanghai Raas Blood, Searainbow Hld Corp, Tonghua Dongbao Pharma, Shanghai Fosun Pharma, Beijing Tongrentang to close down production facilities or suspend expansion into western provinces such as Tibet, Gansu, Xinjiang, Yunnan and Qinghaid due to capital challenges [4]. This in no small way threatens equitable access to quick and affordable pharmaceutical and other healthcare products by the companies as the over 300,000 clinics, pharmacies and hospitals across the Western provinces that get supply from these facilities must resort to supply from other provinces [4].

Moreover, a potential crash in the healthcare market is possible in the healthcare sector as a result of persistent herding tendencies among investors and that may have more damaging consequences for health inequality in China. Consistent with evidence adduced by [14, 35] health sector market crash creates limited supply of healthcare products, black marketeering of strategic healthcare products and unemployment. In China where the healthcare sector employs approximately 12 % of the low income population, the resulting loss of jobs from a health market crash may lead to high cost of healthcare products and services which can fuel income barriers to health equity.

Thus, our findings may explain one of the reason why behavioral economist Richard Thaler thinks that the unsophistication and extrapolation biases in decision among Chinese healthcare investors and companies as well as unguarded response by the government makes a healthcare market crash in China potentially unprecedented in scale [16]. Similarly, the contribution of the Chinese healthcare sector to the global healthcare industry means that any aberrations can have a negative spillover effect and compound the already precarious health inequality in Asia, Africa and countries that substantially depends on China for healthcare services and products.

For example between 2000 and 2014, 34 % of healthcare supplies to member countries of the ASEAN came from China while 57 % percent in Africa came from China. If herding induced market crash leads to a reduction in inflow of medical services, products and equipments to these countries, there is a greater tendency to perpetuate inequity in healthcare in these countries [65]. This stems from the fact that consequential scarcity of healthcare resources will lead to disproportionate allocation of few resources to China dependent countries. Coupled with the expected increase in the cost of the available healthcare resources such as drugs and other supplies (due to scarcity), the resulting high cost of healthcare may likely be monumental as most of these ASEAN and African countries continue to reel under heavy economic challenges at both national and individual level. The effect on global and regional health inequity is thus unlikely to be insignificant.

Consistent with previous studies, these findings elicit serious policy response to ameliorate any potential harmful effect of herding behavior on health equity in both China and other China dependent countries. Similar to the heavy involvement of Chinese government in the business sector, our analysis support the views of [9] of the necessity for the state to provide a greater proportion of public funding for health so that health care provision is not at the peril of private, for profit interests. This will reduce the impact of potential withdrawals of strategic healthcare companies such as Jiangsu Hengrui Med Co, Kangmei Pharmaceutical, Yunnan Baiyao Group Co, Dong E E Jiao Co, Tasly Pharmaceutical, Shanghai Raas Blood, Searainbow Hld Corp, Tonghua Dongbao Pharma, Shanghai Fosun Pharma, Beijing Tongrentang from western provinces deemed unprofitable albeit their healthcare needs.

Inferring from our findings, we are disposed to agree with [27] when they argue that while adequate regulatory framework is necessary to avoid over exploitation by private investors in the healthcare sector in China, some regulatory measures can bring unintended cost and precipitate panic withdrawal and acquisition that stimulates herding behavior and its consequences. In reviewing the literature, we found out that recent pressure on pharmaceutical and healthcare stocks in China following the announcement by the China National Development and Reform Commission ordering price cuts for certain drugs after a government reimbursement scheme for some drugs was note well implemented. This is what led to a sudden hike in the price of healthcare products and subsequently healthcare stocks.

We recommend that future policies can be managed through public-private consensus building and investor sensitization than panic announcements.

## Conclusions and future research direction

Within the limited number of studies on procyclical or herding behavior in the healthcare sector in general and in China in particular, our study sought to find out whether herding syndromes exist among healthcare stock investors and its implication for health equity in China and beyond. We noted the prevalence of herding behavior in almost all the healthcare sector in China using different testing models. This is the first study to compare the effects of different empirical models of herding in the healthcare sector in China in its developing state. More importantly the existence of herding behavior in the health stock market provides significant implication for the healthcare sector and healthcare delivery in China and beyond especially for most countries that depend on China for several medical services and equipments. We have adequately discussed implications of the research for health equity in China and beyond and made policy recommendations. The results inspire curiosity on other remote factors that can trigger disequilibrium in the allocation of healthcare resources in other emerging markets.
